# Same Space, Different Species: The Influence of Exhibit Design on the Expression of Zoo-Housed Apes’ Species-Typical Retiring Behaviors

**DOI:** 10.3390/ani10050836

**Published:** 2020-05-12

**Authors:** Samantha C. Earl, Lydia M. Hopper, Stephen R. Ross

**Affiliations:** Lester E. Fisher Center for the Study and Conservation of Apes, Lincoln Park Zoo, 2001 N Clark Street, Chicago, IL 60614, USA; sam.earl@hotmail.com (S.C.E.); lhopper@lpzoo.org (L.M.H.)

**Keywords:** chimpanzee, exhibit design, gorilla, *Gorilla gorilla*, nesting, *Pan troglodytes*, space use

## Abstract

**Simple Summary:**

Chimpanzees and gorillas in the wild make nests in which to sleep, made from branches, leaves, and other foliage. Chimpanzees typically make their nests in the trees, by bending over branches, whereas gorillas tend to gather materials to form a nest on the ground. We were interested to know if such species patterns in sleeping elevation would be shown by zoo-living apes. We observed a group of six chimpanzees and a group of four gorillas for two 3-month periods in which they inhabited one of two similar indoor/outdoor exhibits, such that each group was observed in both of the exhibits at different times. Specifically, we considered their location while performing retirement behaviors between 3 and 5 p.m. when they settled down for the night. Both species used the exhibits similarly in that in one exhibit, both species nested in elevated locations, whereas in the other, both species tended to nest on the mulch floor. This suggests that particular characteristics of the exhibit spaces affected where and how the apes retired for the night, while the expected species differences that we know from wild populations were not evident. Understanding how the design of zoo exhibits can affect ape behavior will be useful to those seeking to encourage natural patterns of activity.

**Abstract:**

Wild chimpanzees frequently make arboreal nests, while wild lowland gorillas typically nest on the ground. We aimed to understand whether zoo-housed apes’ use of elevated spaces for retiring similarly differed between species and across exhibits. Using a pre-planned exhibit switch at Lincoln Park Zoo (Chicago, USA), we compared where (elevated or terrestrial) two groups of apes (*Pan troglodytes* and *Gorilla gorilla gorilla*) performed retiring behaviors (inactive, sleeping, and nest-building behaviors). We studied a group of six chimpanzees and a group of four gorillas in two exhibits of similar size and configuration for two three-month periods (between 3 and 5 p.m.) before and after the groups switched exhibits. We predicted that chimpanzees would be more likely to retire in elevated locations compared to gorillas, irrespective of the exhibit. We found a significant effect of exhibit on where the apes retired but no effect of species, such that both species were more likely to retire in elevated locations in one exhibit but not the other. This suggests that the specific characteristics of the exhibits (e.g., number of visual barriers) influenced the expression of the apes’ retiring behaviors. These findings offer further insight in how exhibit design can influence the expression of natural behaviors in these species.

## 1. Introduction

Wild chimpanzees (*Pan troglodytes*) and lowland gorillas (*Gorilla gorilla gorilla*) typically spend the majority of their time resting and asleep [1,2]. There is growing recognition that adequately providing for “retiring behaviors”, such as resting, sleeping, and nest-building, is essential for promoting the welfare of captive apes [3]. This reflects the increasing emphasis on the 24/7 welfare approach [4]. One obvious characteristic of ape nests is the elevation at which they are built, and chimpanzees and gorillas typically use different strategies. The nests of wild chimpanzees are most often found high in trees (4.5–24 m) [5,6,7,8], though chimpanzees may make ground nests depending on the local environment [9,10,11]. In contrast, western lowland gorillas typically nest on the ground, especially silverbacks, but will make tree nests if no suitable vegetation is around [8,12,13].

The nesting strategies of wild chimpanzees and lowland gorillas reveal that while both species have a typical strategy (elevated nests for chimpanzees, terrestrial nests for gorillas), these behaviors are plastic and the apes have shown flexibility in response to local conditions [14]. Similarly, in captive settings, chimpanzees and gorillas have demonstrated both elevated and terrestrial sleeping [15,16,17], though the proximate influences on their choices remain largely unknown. To help address this gap in knowledge, we used a pre-planned exhibit switch to study the retirement behaviors of zoo-housed chimpanzees and gorillas in two similar exhibits. 

Our species-by-exhibit design allowed for a direct examination of how exhibit space influences retirement behavior in these apes. We predicted that chimpanzees would prefer to retire in elevated locations, whereas gorillas would prefer to retire on the ground, and that such species differences would remain constant across the exhibits. By growing our understanding of this suite of understudied behaviors, we hope to inform future exhibit design and management practices for these species. 

## 2. Materials and Methods 

### 2.1. Subjects and Housing

We studied two groups of apes housed in separate exhibits at Lincoln Park Zoo, Chicago, IL, USA: a group of six chimpanzees (four females and two males, aged 18–34 years), and an all-male “bachelor” group of four lowland gorillas (aged 11–15 years). All of the apes were captive-born and all but one were mother-reared (one gorilla was hand-reared but had extensive social experience). Both groups lived in the Regenstein Center for African Apes in large indoor/outdoor exhibits, which included deep mulch floors, multiple climbing structures, and sleeping platforms [18], and the apes typically spent 22–23 h a day in their exhibit (i.e., they slept in their exhibits, rather than in a an off-exhibit holding area).

**Figure 1:**
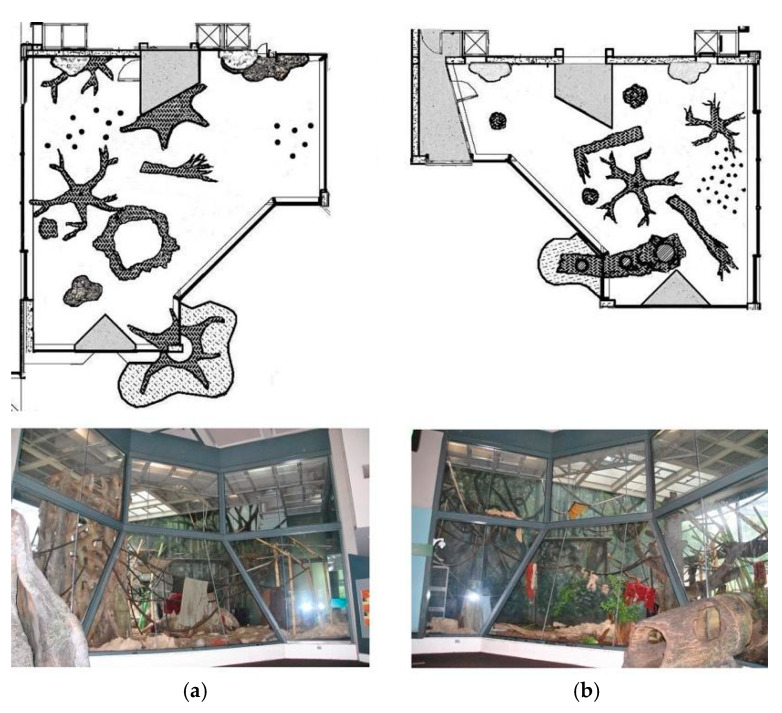
Floor plans and photographs of the two exhibits. (**a**) Exhibit A and (**b**) Exhibit B. Exhibit A has a ground area of 124.5 m^2^ and Exhibit B has a ground area of 99.4 m^2^.

**Figure 2:**
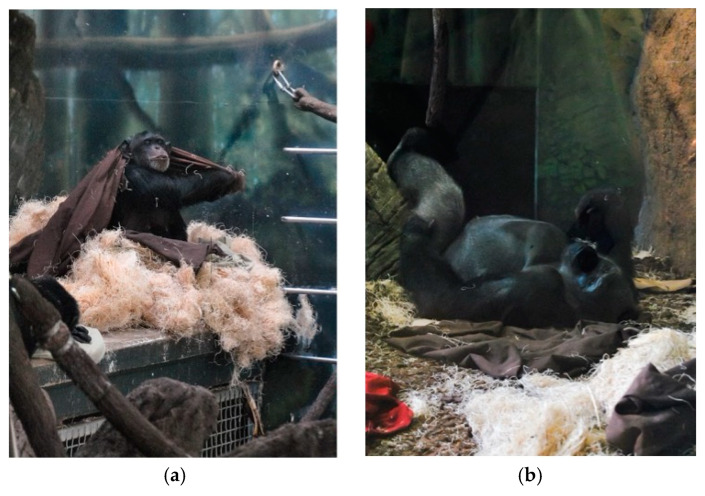
A female chimpanzee creating a nest on an elevated platform in Exhibit B (**a**), and a male gorilla resting terrestrially in Exhibit A (**b**).

We observed the apes in two different indoor/outdoor exhibits—A and B—with both groups spending time in each. Exhibit A and B were similar in design (Figure 1), although the indoor dayroom of Exhibit A was approximately 25% larger than Exhibit B in terms of the ground area, while Exhibit B had modestly (6%) more elevated space than Exhibit A [18]. Both exhibits had sufficient opportunities for the entire group to rest in elevated locations, including flat surfaces at approximately 6 m off the ground and multiple ‘basket-like’ nesters on the wall at approximately 7 m off the ground (Figure 1 and Figure 2). Given the deep mulch floors, and the daily provision of nesting materials (i.e., blankets and wood wool), both groups also had extensive options for terrestrial sleeping in either exhibit space (Figure 1 and Figure 2). 

### 2.2. Protocol

The chimpanzee group was housed in Exhibit A from September 2012 to September 2018, when they were moved to Exhibit B. The gorilla group was housed in Exhibit B from January 2013 (when the group was formed; see [19]) until September 2018, when they were moved to Exhibit A. The exhibits underwent disinfection before the apes entered their new enclosures. We collected behavioral and space-use data on both ape groups for a three-month period, beginning two months after their exhibit switch to allow them an acclimation period (Phase 2: November 2018–January 2019). Additionally, we leveraged our long-term behavioral database to analyze the data collected for the same period the year before the groups switched exhibits (Phase 1: November 2017–January 2018). 

Thus, this study was an opportunistic investigation that took advantage of the planned animal management procedures, and no modifications were made to the standard animal care routines for this study. All the associated data collection and analyses were approved by the Lincoln Park Zoo Research Committee (approval number: 2014-017), which provides oversight for all animal research at the institution. This research adhered to legal requirements in the United States of America and to the American Society of Primatologists’ Principles for the Ethical Treatment of Non-Human Primates.

### 2.3. Data Collection

The data were collected by nine trained observers, including the first author, who had passed the inter-observer reliability tests with 85% agreement, every weekday from 10 a.m. to 5 p.m. We collected the data on Apple^®^ iPad mini^®^ (iOS 9.3.5, Cupertino, CA, USA) using ZooMonitor software (Tracks Software^®^, Salida, CO, USA), developed by Lincoln Park Zoo [20]. Ten-minute focal animal follows, with data recorded every minute, were conducted in Phase 1, and 20-min focal animal follows, with data recorded every two minutes, were conducted in Phase 2. The order of the focal individuals on which the data were collected was randomized each day. The ethogram comprised 48 behaviors and included a second data stream for which the observers recorded the location of the apes. We categorized the individuals as “elevated” when their center of mass was above ground level, excluding small piles of hay or blankets, and “terrestrial” at all other times. Being on elevated substrates included both the elevated locations that the apes used for locomotion (including vines, climbing structures, and cage mesh) as well as those on which the apes rested (including climbing structures, logs, and nesting platforms), though it is notable that the elevated nest sites tended to be in the higher elevations (>6 m). Most of the lower “elevated” sites, such as tree stumps and rockwork, were not suitable for the placement of nests. Furthermore, a previous analysis of vertical space use in this facility showed that both the chimpanzees and gorillas underutilized the intermediate elevated tiers within their exhibits (3–6 m), but used higher elevated tiers (>6 m) as much, or more than expected [21].

### 2.4. Analysis

For our analyses, we focused on a subset of our behavioral data collection and only analyzed those behaviors recorded during the hours of 3 to 5 p.m. The long-term nature of the data collection allowed us to identify the period of time during which the apes were typically settling for the night, and as such, we selected the time window of 3 to 5 p.m. This period followed their last meal of the day and was the period in which most of the nest-building behavior took place. The time at which the apes retired for the night was consistent throughout the year [22] and has been so for many years, likely due to the consistency of the management schedule in the facility. For both species combined, and across both observation phases, this selection criteria resulted in 208 focal follows recorded between 3 and 5 p.m. 

Following Nichols, Kwiatt, Ross, and Hopper [22], we selected the following behaviors from the ethogram as those that represented “retiring behavior” (inactive, nest-building, and sleeping) (Table 1), and focused our analyses on these behaviors. Of the 208 focal follows recorded between 3 and 5 p.m. during our two study phases, 92.31% included one or more observations of the focal animal performing a retirement behavior (all when the apes were in their inside exhibit). Considering just those follows that included retirement behaviors, and to ensure independence across our data points, we analyzed only the first observation of a retirement behavior within a given follow, and we coded each observation of a retirement behavior as either “elevated” (1) or “terrestrial” (0). For Phase 1, this resulted in an average of 9.83 data points per chimpanzee (SD = 1.47) and 10.75 per gorilla (SD = 5.97). Similarly, for Phase 2, this resulted in an average of 8.50 data points per chimpanzee (SD = 3.02) and 9.75 per gorilla (SD = 3.40). 

We used a binomial generalized linear mixed model (GLMM) and fit it using the Laplace approximation via the ‘glmer’ function in the lme4 package in R [23]. We included the subject ID as a random effect, and exhibit and species as fixed effects. We used Akaike Information Criteria (AIC) delta for model selection across three models: one that included species, one that included exhibit, and one that included both factors with an interaction term.

## 3. Results

**Figure 3:**
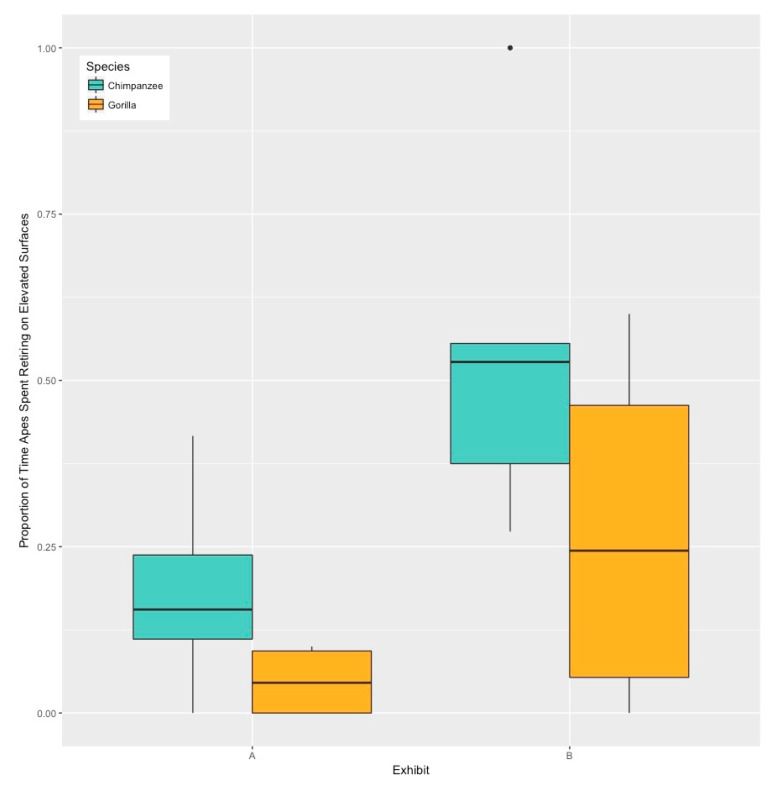
Proportion of time the chimpanzees and gorillas performed retiring behaviors on elevated surfaces in Exhibit A and Exhibit B.

Across both exhibits, the chimpanzees were observed performing retirement behaviors in elevated locations in 35.88% of the observations (SD = 27.11), while the gorillas performed retirement behaviors in elevated locations in 15.99% of the observations (SD = 22.44). However, despite this apparent species difference, the model with the best fit included just the exhibit as a fixed factor (Table 2). This model revealed that the apes were observed performing retirement behaviors in elevated locations significantly more times in Exhibit B than when in Exhibit A (Z = 4.494, *p* < 0.001, 95% CI [1.08, 2.73]) (Figure 3).

## 4. Discussion

The apes’ expression of retirement behaviors, specifically whether they selected elevated or terrestrial locations, was influenced by the exhibit in which they were housed. Both the chimpanzees and gorillas were more likely to perform retirement behaviors in elevated locations when they were in Exhibit B than when they were in Exhibit A. In contrast to our predictions, we did not see a species difference in the location where the apes retired for the night. We attempt here to understand the possible proximate explanations for the observed differences across the exhibits.

For wild apes, a sleeping site choice may be predicated by considerations as simple as choosing a site that will best support the weight of the individual [24,25], or that reflects socially mediated preferences [9]. Environmental factors, such as the presence of predators [6,7,12,26,27] or temperature and humidity (i.e., to aid thermoregulation [10,28]), have also been shown to influence where wild apes choose to sleep. The degree to which the factors that shape nest site selection in wild apes are relevant in captive settings is questionable, but we know that nesting behavior in general is a highly flexible behavior which can come under several potential influences [14]. This flexibility is reflected in our results, in which we saw exhibit- rather than species-driven nesting site choices.

While captive primates do not face predation, fluctuating temperatures is one environmental element common to both wild and captive settings. Lukas et al. [15] discussed thermoregulation as a potential explanation for the high proportion of elevated nests seen among zoo-housed gorillas during colder months, due to the poor thermal properties of traditional concrete substrates. However, the exhibit substrate at Lincoln Park Zoo is a deep mulch bedding, which provides better insulation than concrete. To confirm this benefit, we measured temperatures at different elevations and found that there was not more than a 2 °C difference between the ground level and elevated surfaces in both the exhibits. Another similarity across the ape exhibits at Lincoln Park Zoo is that the elevated surfaces within both were comparably placed relative to the exhibit corners and glass walls that faced the public floor. Thus, given the lack of temperature differences within and between the exhibits, along with the extensive and similar elevated and terrestrial sleeping sites, we tentatively propose that the best explanation for the differences in the retirement location choice across the exhibits that we observed is the differences in (ground-level) visual barriers.

While Exhibit A and B are very similar in size, configuration, and available elevated area, Exhibit A has more visual barriers (i.e., mock termite mounds, larger and more fake tree elements) on the ground level than Exhibit B (Figure 1). These protective elements may provide a sense of security when choosing to nest on the ground [17], similar to that found in captive red-bellied tamarins (*Saguinus labiatus*), which actively selected nest boxes that offered the most concealment [29]. In the wild, western lowland gorillas will avoid nesting in the Gilbertiodendron forest, which has little understory [8,12]; instead, they make most of their terrestrial nests in thick, closed understories. However, if necessary, gorillas will make tree nests in areas with open understories (e.g., primary forests) rather than traveling long distances (200 m) to find an herbaceous nesting site, highlighting their flexibility [8,12,13]. Wild chimpanzees also make tree nests in open understories, but they differ from gorillas in that they prefer this forest type to forage and sleep in and are rarely found nesting in forests with closed understories [8].

Those designing modern zoo exhibits are increasingly considering the natural behaviors of the resident animals. The Regenstein Center for African Apes was the result of comprehensive ape behavior studies in which the apes’ space preferences were measured and integrated into the form and function of the building [18]. However, despite the careful efforts of managers, scientists, and designers, unforeseen exhibit design elements can result in unpredictable impacts on animal behavior. Monitoring and assessing captive animals, to try to ascertain how building design may be influencing their behavior, remains an important strategy—even for those behaviors that are less active or obviously tied to welfare. The degree to which core behaviors, such as resting, nesting, and sleeping, can be influenced by subtle design variations should be taken into consideration in the care and management of great apes and other species.

## 5. Conclusions

The expression of nesting behavior in chimpanzees and gorillas is an important and motivated behavior for those species. Although captive environments differ considerably from those experienced by wild apes, it is worthwhile to consider the factors that may influence the expression of retiring behaviors, such as sleeping and nest-building. In studying both species housed in two similar indoor–outdoor spaces, we found that species differences, predicted by wild behavior, were less likely to predict retiring behavior than were the characteristics of the space in which they lived. The potential of physical environments to influence the expression of animal behavior continues to be of interest to scientists and managers seeking to optimize welfare and care in captive settings. Further research to tease apart the specific characteristics that may be particularly influential will be a required next step in this process.

## Figures and Tables

**Table 1:** The three “retirement” behaviors and their definitions.

Behavior	Definition
Inactive	Focal animal is not moving and not active in any other behavior listed but is not asleep. Behavior includes instances during which a subject holds or carries an object (including food or water), without actively manipulating it (including chewing or swallowing). If the state of wakefulness cannot be determined, the behavior is categorized as “inactive”.
Sleeping	Focal animal is not moving, is not alert, is in a prone position, has eyes closed, and appears to be asleep.
Nest Building	Focal animal manipulates materials in the act of constructing or modifying a nest site. Behavior is limited to modification or immediate construction of the nest itself and does not include gathering and carrying materials with which to nest.

**Table 2:** Generalized linear mixed models (GLMMs) and associated Akaike Information Criteria (AIC) values for the two fixed effects (subject ID was included as a random effect in all the models).

Factors Included in Model	AIC	Model Rank
Exhibit	201.8	1
Exhibit × Species	203.0	2
Species	224.3	3
